# Supplementary material for: Identification and functional analysis of non-coding regulatory small RNA FenSr3 in Bacillus amyloliquefaciens LPB-18
Source: PeerJ. 2023 May 15;11:e15236. doi: 10.7717/peerj.15236 (PMC10194069; doi:10.7717/peerj.15236)
Supplement: Supplemental Information 4 [file peerj-11-15236-s004.zip › KO/CK-vs-T1_map/map01100.html]

KEGG PATHWAY: Metabolic pathways - Reference pathway


|  |  |
| --- | --- |
| **Metabolic pathways - Reference pathway** |  |

[
Pathway menu
| Organism menu
| Pathway entry
|

Hide module list

| User data mapping
|

Image (png) file

]

|  |  |  |
| --- | --- | --- |
| Reference pathway | 100% 70% 35% | 图片下载 |

- **Category title**

  

- **KEGG module**

- Energy metabolism
  - Carbon fixation
    - M00165 Reductive pentose phosphate cycle (Calvin cycle)- M00166 Reductive pentose phosphate cycle, ribulose-5P => glyceraldehyde-3P
      - M00167 Reductive pentose phosphate cycle, glyceraldehyde-3P => ribulose-5P
      - M00168 CAM (Crassulacean acid metabolism), dark
      - M00169 CAM (Crassulacean acid metabolism), light
      - M00172 C4-dicarboxylic acid cycle, NADP - malic enzyme type
      - M00171 C4-dicarboxylic acid cycle, NAD - malic enzyme type
      - M00170 C4-dicarboxylic acid cycle, phosphoenolpyruvate carboxykinase type
      - M00173 Reductive citrate cycle (Arnon-Buchanan cycle)
      - M00579 Phosphate acetyltransferase-acetate kinase pathway, acetyl-CoA => acetate
  - Nitrogen metabolism
    - M00175 Nitrogen fixation, nitrogen => ammonia
  - Methane metabolism
    - M00567 Methanogenesis, CO2 => methane- M00174 Methane oxidation, methanotroph, methane => formaldehyde
  - Sulfur metabolism
    - M00176 Assimilatory sulfate reduction, sulfate => H2S- M00596 Dissimilatory sulfate reduction, sulfate => H2S
      - M00595 Thiosulfate oxidation by SOX complex, thiosulfate => sulfate
- Carbohydrate and lipid metabolism
  - Central carbohydrate metabolism
    - M00001 Glycolysis (Embden-Meyerhof pathway), glucose => pyruvate- M00002 Glycolysis, core module involving three-carbon compounds
      - M00003 Gluconeogenesis, oxaloacetate => fructose-6P
      - M00307 Pyruvate oxidation, pyruvate => acetyl-CoA
      - M00009 Citrate cycle (TCA cycle, Krebs cycle)
      - M00010 Citrate cycle, first carbon oxidation, oxaloacetate => 2-oxoglutarate
      - M00011 Citrate cycle, second carbon oxidation, 2-oxoglutarate => oxaloacetate
      - M00004 Pentose phosphate pathway (Pentose phosphate cycle)
      - M00006 Pentose phosphate pathway, oxidative phase, glucose 6P => ribulose 5P
      - M00007 Pentose phosphate pathway, non-oxidative phase, fructose 6P => ribose 5P
      - M00580 Pentose phosphate pathway, archaea, fructose 6P => ribose 5P
      - M00005 PRPP biosynthesis, ribose 5P => PRPP
      - M00008 Entner-Doudoroff pathway, glucose-6P => glyceraldehyde-3P + pyruvate
      - M00308 Semi-phosphorylative Entner-Doudoroff pathway, gluconate => glycerate-3P
      - M00633 Semi-phosphorylative Entner-Doudoroff pathway, gluconate/galactonate => glycerate-3P
      - M00309 Non-phosphorylative Entner-Doudoroff pathway, gluconate/galactonate => glycerate
  - Other carbohydrate metabolism
    - M00012 Glyoxylate cycle- M00532 Photorespiration
      - M00013 Malonate semialdehyde pathway, propanoyl-CoA => acetyl-CoA
      - M00741 Propanoyl-CoA metabolism, propanoyl-CoA => succinyl-CoA
      - M00632 Galactose degradation, Leloir pathway, galactose => alpha-D-glucose-1P
      - M00552 D-galactonate degradation, De Ley-Doudoroff pathway, D-galactonate => glycerate-3P
      - M00014 Glucuronate pathway (uronate pathway)
      - M00630 D-Galacturonate degradation (fungi)
      - M00631 D-Galacturonate degradation (bacteria)
      - M00061 D-Glucuronate degradation
      - M00081 Pectin degradation
      - M00854 Glycogen biosynthesis, glucose-1P => glycogen/starch
      - M00855 Glycogen degradation, glycogen => glucose-6P
      - M00565 Trehalose biosynthesis, D-glucose 1P => trehalose
      - M00549 Nucleotide sugar biosynthesis, glucose => UDP-glucose
      - M00554 Nucleotide sugar biosynthesis, galactose => UDP-galactose
      - M00761 Undecaprenylphosphate alpha-L-Ara4N biosynthesis, UDP-GlcA => undecaprenyl phosphate alpha-L-Ara4N
  - Fatty acid metabolism
    - M00082 Fatty acid biosynthesis, initiation- M00083 Fatty acid biosynthesis, elongation
      - M00085 Fatty acid biosynthesis, elongation, mitochondria
      - M00415 Fatty acid biosynthesis, elongation, endoplasmic reticulum
      - M00113 Jasmonic acid biosynthesis
      - M00086 beta-Oxidation, acyl-CoA synthesis
      - M00087 beta-Oxidation
      - M00088 Ketone body biosynthesis, acetyl-CoA => acetoacetate/3-hydroxybutyrate/acetone
  - Lipid metabolism
    - M00089 Triacylglycerol biosynthesis- M00098 Acylglycerol degradation
      - M00090 Phosphatidylcholine (PC) biosynthesis, choline => PC
      - M00091 Phosphatidylcholine (PC) biosynthesis, PE => PC
      - M00092 Phosphatidylethanolamine (PE) biosynthesis, ethanolamine => PE
      - M00093 Phosphatidylethanolamine (PE) biosynthesis, PA => PS => PE
      - M00130 Inositol phosphate metabolism, PI=> PIP2 => Ins(1,4,5)P3 => Ins(1,3,4,5)P4
      - M00131 Inositol phosphate metabolism, Ins(1,3,4,5)P4 => Ins(1,3,4)P3 => myo-inositol
      - M00132 Inositol phosphate metabolism, Ins(1,3,4)P3 => phytate
      - M00094 Ceramide biosynthesis
      - M00066 Lactosylceramide biosynthesis
      - M00067 Cerebroside and sulfatide biosynthesis
      - M00099 Sphingosine biosynthesis
      - M00100 Sphingosine degradation
  - Lipopolysaccharide metabolism
    - M00060 Lipopolysaccharide biosynthesis, KDO2-lipid A- M00063 CMP-KDO biosynthesis
      - M00064 ADP-L-glycero-D-manno-heptose biosynthesis
      - M00080 Lipopolysaccharide biosynthesis, inner core => outer core => O-antigen
  - Glycan metabolism
    - M00055 N-glycan precursor biosynthesis- M00072 N-glycosylation by oligosaccharyltransferase
      - M00073 N-glycan precursor trimming
      - M00074 N-glycan biosynthesis, high-mannose type
      - M00075 N-glycan biosynthesis, complex type
      - M00056 O-glycan biosynthesis, mucin type core
      - M00070 Glycosphingolipid biosynthesis, lacto-series, LacCer => Lc4Cer
      - M00071 Glycosphingolipid biosynthesis, neolacto-series, LacCer => nLc4Cer
      - M00068 Glycosphingolipid biosynthesis, globo-series, LacCer => Gb4Cer
      - M00069 Glycosphingolipid biosynthesis, ganglio series, LacCer => GT3
      - M00065 GPI-anchor biosynthesis, core oligosaccharide
  - Glycosaminoglycan metabolism
    - M00057 Glycosaminoglycan biosynthesis, linkage tetrasaccharide- M00058 Glycosaminoglycan biosynthesis, chondroitin sulfate backbone
      - M00059 Glycosaminoglycan biosynthesis, heparan sulfate backbone
      - M00076 Dermatan sulfate degradation
      - M00077 Chondroitin sulfate degradation
      - M00078 Heparan sulfate degradation
      - M00079 Keratan sulfate degradation
  - Terpenoid backbone biosynthesis
    - M00095 C5 isoprenoid biosynthesis, mevalonate pathway- M00096 C5 isoprenoid biosynthesis, non-mevalonate pathway
      - M00364 C10-C20 isoprenoid biosynthesis, bacteria
      - M00365 C10-C20 isoprenoid biosynthesis, archaea
      - M00366 C10-C20 isoprenoid biosynthesis, plants
      - M00367 C10-C20 isoprenoid biosynthesis, non-plant eukaryotes
  - Sterol biosynthesis
    - M00101 Cholesterol biosynthesis, squalene 2,3-epoxide => cholesterol- M00102 Ergocalciferol biosynthesis
      - M00103 Cholecalciferol biosynthesis
      - M00104 Bile acid biosynthesis, cholesterol => cholate/chenodeoxycholate
      - M00106 Conjugated bile acid biosynthesis, cholate => taurocholate/glycocholate
      - M00862 beta-Oxidation, peroxisome, tri/dihydroxycholestanoyl-CoA => choloyl/chenodeoxycholoyl-CoA
      - M00107 Steroid hormone biosynthesis, cholesterol => prognenolone => progesterone
      - M00108 C21-Steroid hormone biosynthesis, progesterone => corticosterone/aldosterone
      - M00109 C21-Steroid hormone biosynthesis, progesterone => cortisol/cortisone
      - M00110 C19/C18-Steroid hormone biosynthesis, pregnenolone => androstenedione => estrone
  - Other terpenoid biosynthesis
    - M00097 beta-Carotene biosynthesis, GGAP => beta-carotene- M00372 Abscisic acid biosynthesis, beta-carotene => abscisic acid
- Nucleotide and amino acid metabolism
  - Purine metabolism
    - M00048 Inosine monophosphate biosynthesis, PRPP + glutamine => IMP- M00049 Adenine ribonucleotide biosynthesis, IMP => ADP,ATP
      - M00050 Guanine ribonucleotide biosynthesis IMP => GDP,GTP
      - M00546 Purine degradation, xanthine => urea
  - Pyrimidine metabolism
    - M00051 Uridine monophosphate biosynthesis, glutamine (+ PRPP) => UMP- M00052 Pyrimidine ribonucleotide biosynthesis, UMP => UDP/UTP,CDP/CTP
      - M00053 Pyrimidine deoxyribonuleotide biosynthesis, CDP/CTP => dCDP/dCTP,dTDP/dTTP
      - M00046 Pyrimidine degradation, uracil => beta-alanine, thymine => 3-aminoisobutanoate
  - Serine and threonine metabolism
    - M00020 Serine biosynthesis, glycerate-3P => serine- M00018 Threonine biosynthesis, aspartate => homoserine => threonine
      - M00555 Betaine biosynthesis, choline => betaine
  - Cysteine and methionine metabolism
    - M00021 Cysteine biosynthesis, serine => cysteine- M00338 Cysteine biosynthesis, homocysteine + serine => cysteine
      - M00609 Cysteine biosynthesis, methionine => cysteine
      - M00017 Methionine biosynthesis, apartate => homoserine => methionine
      - M00034 Methionine salvage pathway
      - M00035 Methionine degradation
  - Branched-chain amino acid metabolism
    - M00019 Valine/isoleucine biosynthesis, pyruvate => valine / 2-oxobutanoate => isoleucine- M00535 Isoleucine biosynthesis, pyruvate => 2-oxobutanoate
      - M00570 Isoleucine biosynthesis, threonine => 2-oxobutanoate => isoleucine
      - M00432 Leucine biosynthesis, 2-oxoisovalerate => 2-oxoisocaproate
      - M00036 Leucine degradation, leucine => acetoacetate + acetyl-CoA
  - Lysine metabolism
    - M00016 Lysine biosynthesis, succinyl-DAP pathway, aspartate => lysine- M00525 Lysine biosynthesis, acetyl-DAP pathway, aspartate => lysine
      - M00526 Lysine biosynthesis, DAP dehydrogenase pathway, aspartate => lysine
      - M00030 Lysine biosynthesis, AAA pathway, 2-oxoglutarate => 2-aminoadipate => lysine
      - M00433 Lysine biosynthesis, 2-oxoglutarate => 2-oxoadipate
      - M00031 Lysine biosynthesis, mediated by LysW, 2-aminoadipate => lysine
      - M00032 Lysine degradation, lysine => saccharopine => acetoacetyl-CoA
  - Arginine and proline metabolism
    - M00015 Proline biosynthesis, glutamate => proline- M00028 Ornithine biosynthesis, glutamate => ornithine
      - M00763 Ornithine biosynthesis, mediated by LysW, glutamate => ornithine
      - M00844 Arginine biosynthesis, ornithine => arginine
      - M00845 Arginine biosynthesis, glutamate => acetylcitrulline => arginine
      - M00029 Urea cycle
  - Histidine metabolism
    - M00026 Histidine biosynthesis, PRPP => histidine- M00045 Histidine degradation, histidine => N-formiminoglutamate => glutamate
  - Aromatic amino acid metabolism
    - M00022 Shikimate pathway, phosphoenolpyruvate + erythrose-4P => chorismate- M00023 Tryptophan biosynthesis, chorismate => tryptophan
      - M00024 Phenylalanine biosynthesis, chorismate => phenylalanine
      - M00025 Tyrosine biosynthesis, chorismate => tyrosine
      - M00040 Tyrosine biosynthesis, prephanate => pretyrosine => tyrosine
      - M00042 Catecholamine biosynthesis, tyrosine => dopamine => noradrenaline => adrenaline
      - M00043 Thyroid hormone biosynthesis, tyrosine => triiodothyronine/thyroxine
      - M00044 Tyrosine degradation, tyrosine => homogentisate
      - M00350 Capsaicin biosynthesis, L-Phenylalanine => Capsaicin
      - M00037 Melatonin biosynthesis, tryptophan => serotonin => melatonin
      - M00038 Tryptophan metabolism, tryptophan => kynurenine => 2-aminomuconate
  - Other amino acid metabolism
    - M00033 Ectoine biosynthesis, aspartate => ectoine- M00027 GABA (gamma-Aminobutyrate) shunt
      - M00047 Creatine pathway
  - Cofactor and vitamin biosynthesis
    - M00127 Thiamine biosynthesis, AIR => thiamine-P/thiamine-2P- M00125 Riboflavin biosynthesis, GTP => riboflavin/FMN/FAD
      - M00124 Pyridoxal biosynthesis, erythrose-4P => pyridoxal-5P
      - M00115 NAD biosynthesis, aspartate => NAD
      - M00119 Pantothenate biosynthesis, valine/L-aspartate => pantothenate
      - M00120 Coenzyme A biosynthesis, pantothenate => CoA
      - M00572 Pimeloyl-ACP biosynthesis, BioC-BioH pathway, malonyl-ACP => pimeloyl-ACP
      - M00123 Biotin biosynthesis, pimeloyl-ACP/CoA => biotin
      - M00573 Biotin biosynthesis, BioI pathway, long-chain-acyl-ACP => pimeloyl-ACP => biotin
      - M00577 Biotin biosynthesis, BioW pathway, pimelate => pimeloyl-CoA => biotin
      - M00126 Tetrahydrofolate biosynthesis, GTP => THF
      - M00840 Tetrahydrofolate biosynthesis, mediated by ribA and trpF, GTP => THF
      - M00841 Tetrahydrofolate biosynthesis, mediated by PTPS, GTP => THF
      - M00842 Tetrahydrobiopterin biosynthesis, GTP => BH4
      - M00843 L-threo-Tetrahydrobiopterin biosynthesis, GTP => L-threo-BH4
      - M00121 Heme biosynthesis, glutamate => heme
      - M00846 Siroheme biosynthesis, glutamate => siroheme
      - M00129 Ascorbate biosynthesis, animals, glucose-1P => ascorbate
      - M00114 Ascorbate biosynthesis, plants, glucose-6P => ascorbate
      - M00550 Ascorbate degradation, ascorbate => D-xylulose-5P
      - M00112 Tocopherol/tocotorienol biosynthesis
      - M00122 Cobalamin biosynthesis, cobinamide => cobalamin
      - M00117 Ubiquinone biosynthesis, prokaryotes, chorismate => ubiquinone
      - M00128 Ubiquinone biosynthesis, eukaryotes, 4-hydroxybenzoate => ubiquinone
      - M00116 Menaquinone biosynthesis, chorismate => menaquinone
      - M00118 Glutathione biosynthesis, glutamate => glutathione
      - M00140 C1-unit interconversion, prokaryotes
      - M00141 C1-unit interconversion, eukaryotes
  - Polyamine biosynthesis
    - M00133 Polyamine biosynthesis, arginine => agmatine => putrescine => spermidine- M00134 Polyamine biosynthesis, arginine => ornithine => putrescine
      - M00135 GABA biosynthesis, eukaryotes, putrescine => GABA
      - M00136 GABA biosynthesis, prokaryotes, putrescine => GABA
- Secondary metabolism
  - Aromatics degradation
    - M00537 Xylene degradation, xylene => methylbenzoate- M00551 Benzoate degradation, benzoate => catechol / methylbenzoate => methylcatechol
      - M00568 Catechol ortho-cleavage, catechol => 3-oxoadipate
      - M00569 Catechol meta-cleavage, catechol => acetyl-CoA / 4-methylcatechol => propanoyl-CoA
      - M00544 Carbazole degradation, carbazole => 2-oxopent-4-enoate + anthranilate
      - M00418 Toluene degradation, anaerobic, toluene => benzoyl-CoA
      - M00541 Benzoyl-CoA degradation, benzoyl-CoA => 3-hydroxypimeloyl-CoA
      - M00534 Naphthalene degradation, naphthalene => salicylate
      - M00624 Terephthalate degradation, terephthalate => 3,4-dihydroxybenzoate
      - M00623 Phthalate degradation, phthalate => protocatechuate
  - Biosynthesis of secondary metabolites
    - M00039 Monolignol biosynthesis, phenylalanine / tyrosine => monolignol- M00137 Flavanone biosynthesis, phenylalanine => naringenin
      - M00138 Flavonoid biosynthesis, naringenin => pelargonidin
      - M00661 Paspaline biosynthesis, geranylgeranyl-PP + indoleglycerol phosphate => paspaline
